# New Neotropical Sebacinales Species from a *Pakaraimaea dipterocarpacea* Forest in the Guayana Region, Southern Venezuela: Structural Diversity and Phylogeography

**DOI:** 10.1371/journal.pone.0103076

**Published:** 2014-07-29

**Authors:** Bernard Moyersoen, Michael Weiß

**Affiliations:** 1 School of Biological Sciences, Cruickshank Building, University of Aberdeen, Aberdeen, United Kingdom; 2 Department of Biology, University of Tübingen, Tübingen, Germany; University of Florida, United States of America

## Abstract

*Pakaraimaea dipterocarpacea*, a member of the Dipterocarpaceae endemic in the Guayana region, is associated with a diverse community of ectomycorrhizal (ECM) fungi. Amongst the 41 ECM fungal species detected in a 400 m^2^ *P. dipterocarpacea* ssp. *nitida* plot in Southern Venezuela, three species belonged to the Sebacinales. We tested whether ECM anatomotype characterization can be used as a feasible element in an integrative taxonomy in this diverse fungal group, where the relevance of fruitbody morphology for species delimitation seems limited. Using a combination of ECM morpho-anatomical characterizations and phylogenetic analyses based on nuclear ITS and LSU sequences, we report three new species. The main distinguishing features of *Sebacina guayanensis* are the yellowish cell walls together with conspicuous undifferentiated, uniform compact (type B) rhizomorphs. Staghorn-like hyphae are characteristic of *S. tomentosa*. The combination of clusters of thick-walled emanating hyphae, including hyphae similar to awl-shaped cystidia with basal dichotomous or trichotomous ramifications, and the presence of type B rhizomorphs were characteristic of a third, yet unnamed species. The three species belong to three different, possibly specifically tropical clades in Sebacinales Group A. The geographic distribution of phylogenetically related strains was wide, including a *Dicymbe* forest in Guyana and an Ecuadorian rainforest with *Coccoloba* species. We show that ECM morpho-anatomy can be used, in combination with other analyses, to delineate species within Sebacinales Group A. In addition to phylogenetic information, type B rhizomorphs observed in different Sebacinales clades have important ecological implications for this fungal group. The phylogeography of Sebacinales suggests that dispersion and host jump are important radiation mechanisms that shaped *P. dipterocarpacea* ECM fungal community. This study emphasizes the need for more sequence data to evaluate the hypothesis that phylogeographic relationships between neo- and paleotropical ECM fungal species could be attributed to the vicariance of cross-continental hosts such as the Dipterocarpacae.

## Introduction

Sebacinales is a genetically diverse group of basidiomycetes with a broad range of lifestyles including a diversity of mycorrhizal and endophytic associations [1]. These fungi are widespread and the same species can colonize a wide range of host plants and be involved in different symbiotic associations [1], [2]. Molecular phylogenetic studies have shown that Sebacinales are divided in two groups, informally called A and B [3]. Up to date, most ectomycorrhizal (ECM) associations have been documented from Group A [1], [3] and only one clade in Group B included ECM strains [4]. Several recent studies mostly based on DNA surveys indicate that Sebacinales are frequently involved in ECM across the tropics [5], [6]. No clear patterns in the biogeography of these tropical ECM Sebacinales have been observed yet [6], although no phylogeographic study with an emphasis on tropical ECM Sebacinales has been published to date.

There is a general view that fruitbody morphology is of limited use for the description of ECM Sebacinales diversity. Many fruitbodies produced by Sebacinales involved in ECM are inconspicuous and these structures have obviously developed independently several times [3], [7]. Septal pore ultrastructure is generally recognized as a characteristic (yet not unique) feature of Sebacinales hyphae involved in different types of associations with plant roots (e.g., [3]).

Fungal species can be distinguished based on ECM anatomy [8]. In general, ECM anatomotypes in various fungal lineages have mostly been described from temperate ecosystems and there is a need to improve the knowledge about tropical ECM morpho-anatomy [9]. Despite the great diversity of Sebacinales strains reported to date [1], [10], only ten Sebacinales ECM anatomotypes have been described precisely worldwide and only three of them are from tropical tree species [5].

This study is part of a phylogeographic and taxonomic survey of ECM fungi associated with *Pakaraimaea dipterocarpacea* ssp. *nitida* Maguire & Steyerm. in Southern Venezuela [5], [11], [12], [13], [14], [15]. *Pakaraimaea dipterocarpacea*, an endemic tree species from the Guayana region [11], [16], [17], is one of the few known locally dominant ECM tree species in the neotropical lowland forests. This tree belongs to the Dipterocarpaceae, one of the most important ECM tropical tree family in SE Asia [18]. The distribution of Dipterocarpaceae s.l. has been attributed to a Gondwanan origin of this plant family [16], [19], [20], although this hypothesis has been challenged by molecular clock studies [21]. The disjunct distribution of *P. dipterocarpacea* suggests that ancestors of Dipterocarpaceae acquired the capacity to associate with ECM fungi before the splitting of South America from Gondwana [11]. There are alternative proposals to explain the ECM status of *P. dipterocarpacea*, such as an independent acquisition of ECM association in South America or a more recent transoceanic long-distance dispersal of ECM Dipterocarpaceae to the neotropics [22]. The phylogeography of *P. dipterocarpacea* ECM fungi might reflect the disjunct distribution of this endemic tree species. A diverse ECM fungal community was observed in a 400 m^2^ *P. dipterocarpacea* forest plot [12]. In total, 41 ECM fungal species (including an unpublished strain belonging to *Elaphomyces*) were reported and up to twelve different ECM anatomotypes were found to colonize a single 125 cm^3^ soil core [12]. ECM fungal DNA comparisons between forests in the Guayana region and a molecular phylogenetic analysis of two newly reported *Inocybe* species [12], [15] suggested that host jump and fungal dispersion, i.e., long-distance dispersal across a pre-existing barrier [23], were important radiation mechanisms involved in shaping the fungal community of *P. dipterocarpacea* [12]. ECM fungi possibly endemic in the forest indicated that the *P. dipterocarpacea* fungal community might also include old relictual species [12]. Similar trends regarding the great ECM fungal diversity, the dominance of host-generalist fungi and the presence of possibly endemic Agaricales were observed in a *P. dipterocarpacea* ssp. *dipterocarpacea* forest in Guyana [24].

Sebacinales were selected to extend our study on phylogeography of *P. dipterocarpacea* ECM fungi. *Tremellodendron ocreatum* and *S*. *incrustans* fruitbodies were first described in a *Dicymbe* (Fabaceae/Caesalpinioideae) rain forest in the Guayana region [25]. The first report of Sebacinales ECM in the tropics was made on *P. dipterocarpacea* [11]. Subsequently, tropical Sebacinales ECM strains were recorded from DNA samples from across the tropics [12], [26], [27], [28], [29], [30], [31], [32], [33], [34], [35], [36], [37]. In total, three different Sebacinales strains (specimens BM03M3, PD10 and 6MM2) were found in the *P. dipterocarpacea* forest plot studied [12]. The first detailed descriptions of tropical ECM anatomotypes involving Sebacinales were made from these specimens [5]. That study showed that specimens BM03M3, PD10 and 6MM2 could be clearly distinguished on the basis of morpho-anatomical characters. Further combined morpho-anatomical and molecular phylogenetic studies were necessary in order to decide whether these three newly described *P. dipterocarpacea* Sebacinales ECM anatomotypes belonged to species already known to science or whether it would be appropriate to describe new species for them.

For the present study, we performed morpho-anatomical descriptions of several specimens similar to BM03M3, PD10 and 6MM2 in combination with molecular phylogenetic analyses. The degree of host specificity and the phylogeography of these three *P. dipterocarpacea* Sebacinales species were analyzed with a broad array of taxa including Sebacinales strains collected worldwide.

## Materials and Methods

### Study site

All collections were from a 400 m^2^ plot [11], [12], located at 4°20′N, 61°48′W, altitude 500 m, near Icabarú Village, in Gran Sabana, Estado Bolivar, Venezuela. The study site was selected based on a previous report of a stand of *P. dipterocarpacea* ssp. *nitida* in the same area [38]. A second ECM tree species, *Aldina* sp. (Fabaceae/Faboideae), also occurs in the plot [11]. No specific permissions were needed for this study which is located outside a protected area. This study was made in collaboration with the Venezuelan Institute for Scientific Research (IVIC) (G Cuenca) and University Simon Bolivar (T Iturriaga). Both G Cuenca and T Iturriaga are supported by the Venezuelan government research institution and are legally authorized to perform this study. No protected species were sampled.

### Root harvest and morpho-anatomical descriptions

Root samples were collected in November 2003, July 2007 and 2008 (Table 1). The sampling procedures are described in ref. [5] and ref. [12]. All Sebacinales ECM specimens were sampled in top organic soil layer, below the litter, either by root tracing or in nine 125 cm^3^ soil cores. Morphology of traced *P. dipterocarpacea* and *Aldina* sp. fresh secondary roots was characterized for subsequent putative host identification of ECM from the soil cores. ECM were washed from the soil and clusters were assigned to morphotypes using a field dissecting microscope or a 10X magnifying glass. ECM tips were stored in 2% cetyltrimethylammonium bromide (CTAB). Depending on the available ECM material, subsamples from the same cluster were also fixed in formaldehyde: acetic acid: 70% ethanol (5∶5∶90) (FEA), glycerol: 100% ethanol: water (1∶1∶1) or water at 5°C. The morpho-anatomy of undescribed ECM specimens was compared with BM03M3, PD10 and 6MM2 [5] anatomotypes, following Agerer's [39] method of description. Descriptions were made using a Normarski interference contrast microscope fitted with a drawing tube and a digital eyepiece, at a magnification of X1000. Reference specimens are stored in the University of Liège Herbarium (LG).

**Table 1:** Collections and accession numbers of Sebacinales ectomycorrhizal samples collected in the 400^2^ *Pakaraimaea dipterocarpacea* plot.

Ectomycorrhizal sample	Sampling date	Location	Sampling procedure	Host§	Anatomotype¥	GenBank access. nos
BM03M3	25 Nov. 2003	Venezuela, 4°20′N, 61°48′W	Root tracing	P. dipterocarpacea*	BM03M3^+^	KF773775
PD51	17 July 2007	Venezuela, 4°20′N, 61°48′W	Root tracing	P. dipterocarpacea*	Like BM03M3^++^	KF773776
BM07M6	19 July 2007	Venezuela, 4°20′N, 61°48′W	Soil core	Unknown	Like BM03M3^++^	KF773777
5MM2	27 July 2008	Venezuela, 4°20′N, 61°48′W	Soil core	Unknown	Like BM03M3^++^	KF773778
PD10	20 July 2007	Venezuela, 4°20′N, 61°48′W	Root tracing	*P. dipterocarpacea*	PD10^+++^	KF773779
PD91	17 July 2007	Venezuela, 4°20′N, 61°48′W	Root tracing	*P. dipterocarpacea*	Like PD10^+++^	KF773780
1AM1	24 July 2008	Venezuela, 4°20′N, 61°48′W	Soil core	*Aldina* sp.**	Like PD10^+++^	KF773781
10PDM3	31 July 2008	Venezuela, 4°20′N, 61°48′W	Soil core	P. dipterocarpacea***	Like PD10^+++^	KF773782
6MM2	31 July 2008	Venezuela, 4°20′N, 61°48′W	Soil core	P. dipterocarpacea**	6MM2^+++^	KF773783

Vouchers of BM03M3 and PD10 specimens are deposited in University of Liège Herbarium (LG).

§ : EcM Host identity confirmed after *rbc*L RFLP matching* or sequencing**; Putative host identification from secondary root morphology***.

¥ : Fixation of ECM subsample for further anatomical description: glycerol: 100% ethanol: water (1∶1∶1)^+^, formaldehyde: acetic acid: 70% ethanol (5∶5∶90) (FEA) ^++^ or 2% cetyltrimethylammonium bromide^+++^.

### DNA protocols

Methods for DNA extraction, amplification and sequencing were described in ref. [11] and ref. [12]. Genomic DNA was extracted using a DNeasy Plant Kit (Qiagen S.A. Courtaboeuf, France). The ITS and partial LSU regions of the nuclear rDNA repeat were PCR-amplified with forward primer ITS1f in combination with LR6 [40], [41]. If multiple or no PCR products were obtained, DNA was extracted from another ECM tip of the same cluster, or the same extract was reamplified using the following primers in different combinations: ITS1f, ITS4b, ITS4, 5,8SR, LR21, LROR, LR6 ([40], [41], [42], R Vilgalys Lab http://www.biology.duke.edu/fungi/mycolab/primers.htm). To check host identity, *rbc*L DNA was amplified from ECM samples BM03M3, 6MM2 [12] and 1AM1 using the primers *rbc*LN and *rbc*LR [43], following the PCR protocols described in ref. [11]. Sequencing was performed either by the Genotranscriptomics Platform, GIGA, University of Liège, or on an automated sequencer (ABI 3130xl; Applied Biosystems) in the Systematic Botany and Mycology Group, University of Tübingen, using the same primers as for PCR. Sequence editing was performed using Sequencher, version 4.0 (Gene Codes Corporation). ITS and LSU sequences generated in this study have been deposited at the National Center for Biotechnology Information (NCBI, GenBank: http://www.ncbi.nlm.nih.gov) under accession numbers KF773775-773783 (Table 1).

### Phylogenetic analyses

For the phylogenetic analyses of ITS and LSU sequences, a set of reference sequences from GenBank and UNITE [44] was built as follows. First, the closest matches for both ITS and LSU sequences from BM03M3, PD10 and 6MM2 anatomotypes were retrieved from GenBank using BLASTn [45]. To include in the data set all published tropical ITS and LSU sequences of Sebacinales strains expected to be involved in ECM association, a total of 99 ITS and 52 LSU tropical Sebacinales sequences including 34 ITS-only sequences were recovered from GenBank and UNITE, based on a literature survey. For molecular phylogenetic comparisons with published Sebacinales anatomotypes, all available ITS and LSU sequences from members of the Sebacinales with a described ECM morphology and anatomy were also included in the data set. To eliminate suspicious or chimeric sequences, highly divergent sequences detected in a preliminary RAxML phylogenetic analysis (see below) were submitted to BLASTn search against the GenBank nucleotide database. After elimination of obviously chimeric sequences and replicate sequences from a preliminary alignment, the reference data set obtained contained 159 Sebacinales sequences. A total of 74 of these sequences (including the 9 specimens from *P. dipterocarpacea* forest) were from a tropical origin. The following sequence sampling variants were used in subsequent analyses: the reference data set together with four sequences belonging to BM03M3, PD10 and 6MM2 anatomotypes (data set A); data set A together with five sequences from specimens belonging to BM03M3 and PD10 anatomotypes (data set B); data set A without the two particularly short tropical ITS sequences, FN669398 (181 bp) and FN557561 (131 bp) (data set C). To facilitate the alignment of sequences including ITS, LSU or both ITS and LSU regions, the data sets were split into ITS and LSU sequences components before alignment.

To align the ITS and LSU sequences with a wide range of Sebacinales sequences including strains with different trophic status and belonging to both Groups A and B, ITS and LSU data set components were aligned with a profile alignment analysed in ref. [4], using the seed option of MAFFT 6.903b [46], [47]. Alignments were performed separately for ITS and LSU regions and the two resulting alignments were then concatenated. Minimal manual adjustments were performed using Bioedit on leading or trailing regions. The same alignment strategy was performed for each data set variant (A, B, C).

Phylogenetic analyses of the integrated ITS and LSU data were performed using a maximum likelihood (ML) approach involving starting trees obtained by 1000 replicates of rapid bootstrapping in RAxML version 7.3.1 [48] using the GTRCAT model for DNA substitution. A preliminary phylogenetic analysis of data set A aligned to the complete profile alignment used in ref. [4] was performed to confirm the positions of BM03M3, PD10 and 6MM2 together with the remaining published anatomotypes in Sebacinales Group A. To reduce sampling size, only five Group B sequences belonging to the *Sebacina vermifera* complex (AY505548, DQ983816, DQ520096, AY505555) and *Piriformospora indica* (AY505557) were maintained in the alignment. An approach identical to the preliminary analysis was performed for the phylogenetic analyses of each data set variant (A, B, C). The alignment of data set B included 444 Sebacinales sequences and 1585 nucleotide positions. The best tree inferred in each phylogenetic analysis including bootstrap values was displayed and edited using FigTree v. 1.3.1 [49]. The trees were rooted with the five Sebacinales Group B sequences.

### Nomenclature

The electronic version of this article in Portable Document Format (PDF) in a work with an ISSN or ISBN will represent a published work according to the International Code of Nomenclature for algae, fungi, and plants, and hence the new names contained in the electronic publication of a PLoS ONE article are effectively published under that Code from the electronic edition alone, so there is no longer any need to provide printed copies. In addition, new names contained in this work have been submitted to MycoBank and Index Fungorum from where they will be made available to the Global Names Index. The unique MycoBank numbers can be resolved and the associated information viewed through any standard web browser by appending the MycoBank numbers contained in this publication to the prefix http://www.mycobank.org/MB/. The online version of this work is archived and available from the following digital repositories: PubMed Central (www.ncbi.nlm.nih.gov/pubmed) and LOCKSS (www.lockss.org/).

## Results

### Species descriptions

#### *Sebacina guayanensis* B. Moyersoen & M. Weiß, sp. nov. MycoBank MB808460; Index Fungorum IF550251 (Figs. 1–2; ref. [5], Figs. 1–8)

*Typus*: Venezuela, 4°20′N, 61°48′W, near Icabarú, in the Caroní headwaters, Estado Bolivar, leg. B. Moyersoen, 25 Nov. 2003, ECM collected by root tracing from a *P. dipterocarpacea* ssp. *nitida* Maguire & Steyerm. adult tree, specimen in glycerol/ethanol/water, B. Moyersoen BM03M3, in LG.

**Figure 1:**
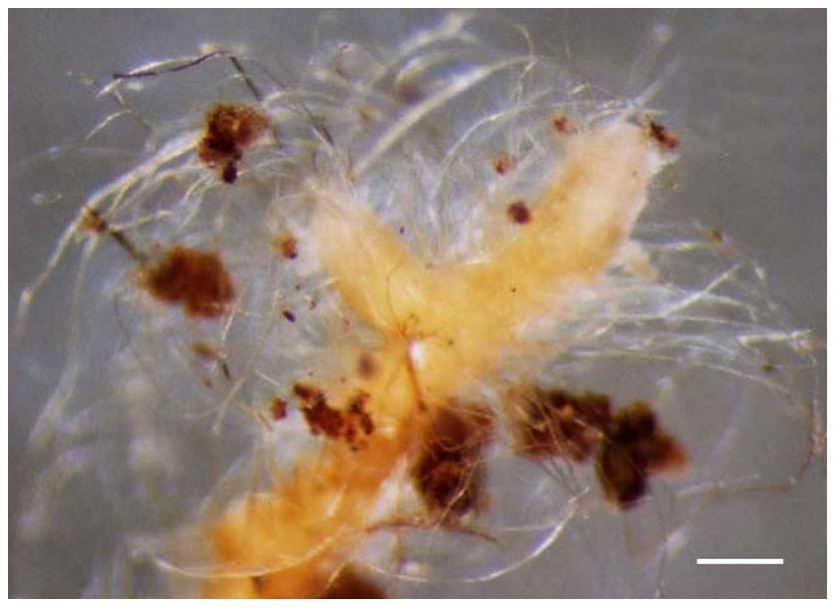
*Sebacina guayanensis* + *Pakaraimaea dipterocarpacea* ssp. *nitida*. Habit of ectomycorrhiza, woolly surface, emanating hyphae and rhizomorphs occurring throughout, soil particles sticking to the mantle and rhizomorphs surface. Bar  =  0.3 mm. (Fig. from specimen BM03M3).

**Figure 2:**
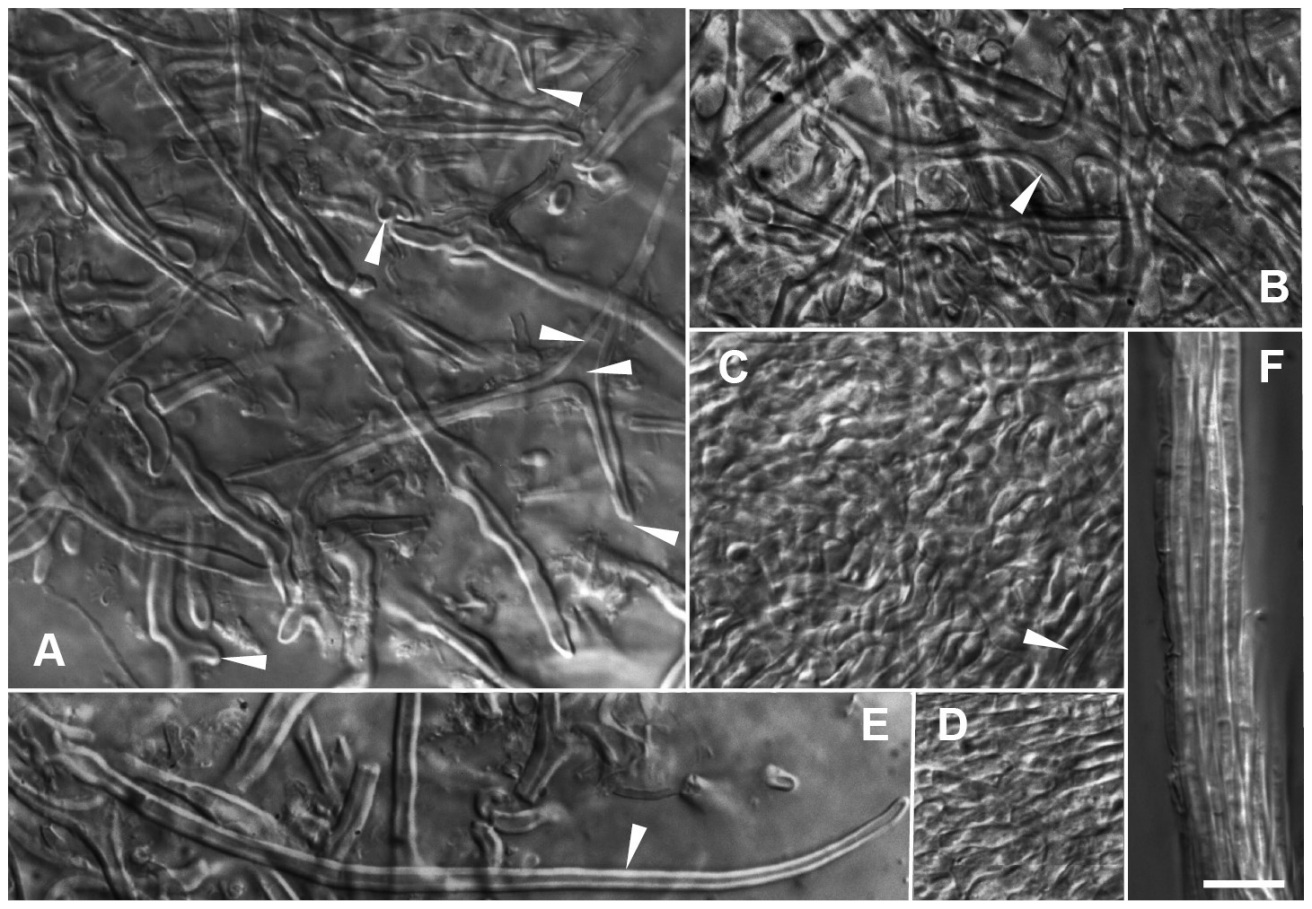
*Sebacina guayanensis* + *Pakaraimaea dipterocarpacea* ssp. *nitida*. – A–C. Plan views of outer layer. – A–B. Plan views of hyphal net in outer mantle layer. Ramified, thick-walled hyphae, triangular inflations at ramifications (arrow-head), short protrusions (arrow-heads), aculeate distal hyphal ends (arrow-heads), clampless hyphae (arrow-head) (A). Polytomies with three branches (arrow-head) (B). – C. Plan view of outer layer below the net, transition between a dense plectenchyma of thick-walled ramified hyphae and a pseudoparenchyma, with a matrix (arrow-head). – D. Plan view of middle mantle layer, a dense plectenchyma of ramified hyphae. – E. Emanating hypha similar to awl shaped cystidium (arrow-head). – F. Rhizomorph, thick walled hyphae of uniform diameter running in parallel. Bar  =  10 µm. (Fig. 1-A, C-F from specimen BM03M3 and 1-B from BM07M6).

*Short description*: The yellowish, woolly ECM of *S. guayanensis* are characterized anatomically by the combination of yellowish or light orange hyphal walls and the conspicuous uniformly compact type B rhizomorphs. Cells in the net covering the outer ectomycorrhizal mantle layer are 2.3-3.0-3.7 µm diam. and the outer mantle layer is a transition between a dense plectenchyma with ramified hyphae and a pseudoparenchyma, with a matrix. Polytomies with three branches are present in the net. Hyphae emanating from the mantle are 2.7-3-3.3 µm diam. and some are similar to awl-shaped cystidia. Cells in the rhizomorphs are 2.5-2.7-3.5 µm in diam. Cell walls are thick in outer mantle layer, rhizomorphs and emanating hyphae. Nuclear rDNA data clearly support the separation of this species from other known species in the group.

*Etymology*: Epithet *guayanensis* relating to the record of this new species in the Venezuelan Guayana region.

*Description of the species*: *Ectomycorrhizal systems* yellowish (G in Colour Identification Chart, Royal Botanic Garden Edinburgh, Flora of British Fungi, 1969) in older parts, white at apex, with a woolly surface; emanating hyphae frequent, occurring throughout, colourless; rhizomorphs frequent, occurring throughout, colourless or yellowish; mycorrhizas hydrophilic, belonging to a transition between a short distance and a smooth subtype of medium distance *exploration type*. *Outer ectomycorrhizal mantle layer*, a transition between a dense plectenchyma with thick-walled ramified hyphae and a pseudoparenchyma (type A/C/H, ref. [50]), with a matrix, covered by a loose net of ramified, thick-walled hyphae from which emanating hyphae and rhizomorphs originate; hyphae from the net mostly cylindrical, with frequent short protrusions and inflations including triangular inflations at ramifications, and aculeate distal ends; sometimes polytomies with three branches; cells 2.3-3.0-3.7 µm diam.; cell walls light orange, smooth, irregularly thick (0.5-1-1.7 µm); hyphae deeper in surface layer variably shaped, with walls difficult to distinguish from the matrix; walls and matrix yellowish; short hyphal protrusions present in the outer mantle layer and the net. *Deeper ectomycorrhizal mantle layer*, a dense plectenchyma of ramified cylindrical hyphae with thin, smooth, yellowish walls. *Rhizomorphs* uniform, compact (with uniform, thick-walled hyphae, densely packed and glued together, type B in ref. [50]), 5–25 µm diam., often with ramified hyphae at distal end; hyphae 2.5-2.7-3.5 µm diam.; walls smooth, yellowish, 0.7-1-1.5 µm thick, thin at distal end. *Emanating hyphae* smooth, yellowish, 2.7-3-3.3 µm diam., with or without short protrusions at proximal end; hyphal end simple; walls smooth, yellowish, 0.5-1-1.5 µm thick, sometimes thinner at distal end; ramifications lacking; some emanating hyphae similar to awl-shaped cystidia. *Anastomoses* open [8]. *Clamps* absent, septa frequent, generally straight; septal pores of dolipore parenthesome type; parenthesomes imperforate, with electron-dense middle lamella. *Mantle preparation* slightly dextrinoid. *Hartig net* paraepidermical.

*Material studied*: Venezuela, 4°20′N, 61°48′W, near Icabarú, in the Caroní headwaters, Estado Bolivar, leg. B. Moyersoen, 25 Nov. 2003, ECM collected by root tracing from a *P. dipterocarpacea* ssp. *nitida* Maguire & Steyerm. adult tree, specimen in glycerol/ethanol/water, holotype designated as B. Moyersoen BM03M3 in LG. Further material studied: leg. B. Moyersoen, 17 July 2007, mantle preparation in lactic acid (90%) in Herb. B. Moyersoen PD51; leg. B. Moyersoen, 19 July 2007, ECM collected near a *P. dipterocarpacea* tree, below an *Amanita* sp. (BM07C21) fruiting body, specimen in FEA in Herb. B. Moyersoen BM07M6; leg. B. Moyersoen, 27 July 2008, specimen in CTAB in Herb. B. Moyersoen 5MM2.

*Molecular characters*: The consensus sequence from three strains (BM03M3, PD51, 5MM2) of *S. guayanensis* sequenced in this study is given in the following format: [3′ 18S]ITS1*5.8S*ITS2[5′ 28S]. Nucleotides in bold indicate differences among sequences: underlined positions are polymorphic between the studied specimens, R = A/G, K = G/T).

[CATTA]ATGAGTGTGAATCGTTGCCTTTGGTGCTGGCTCCGGCATGTGCACACTGGTGACTTTCATCCAATACCCTCGTGAACCCTTTGCCTCTTGCTGGCTTTGGCTGGCAGAGGATTTTATACACTCACTCGAATGTAATGAAACCTATTATTGTGCACAAGCACTAATGTAC*AACTTTCAACAACGGATCTCTTGGCTCTCGCATCGATGAAGAACGCAGCGAAATGCGATAAGTAATGTGAATTGCAGAATTCAGTGAATCATCGAATCTTTGAACGCACCTTGCACCCTTTGGAATTCCGAAGGGTATGCTCGTTTGAGTGTCAT*TGTACTCTCACACTCTTCAATCA**R**TTGGATTTGGGGAGTGGTGGACTTGGGTGTTGCCGCTTTACTGTGGCTCACCTTAAATGCTTTAGTGCAACTCTTAGTTAGACATAGTACGGCGTGATAAGTATCTTCGCCGGCACCTTGCATGAGGGTGGCTAATTGAGAGC**K**CTGTGCTTCAAACTGTCTTCGGACAATCTCTGACAACTTGGGCCTCAAAT[CGAGTAGGACT]

**Figure 3:**
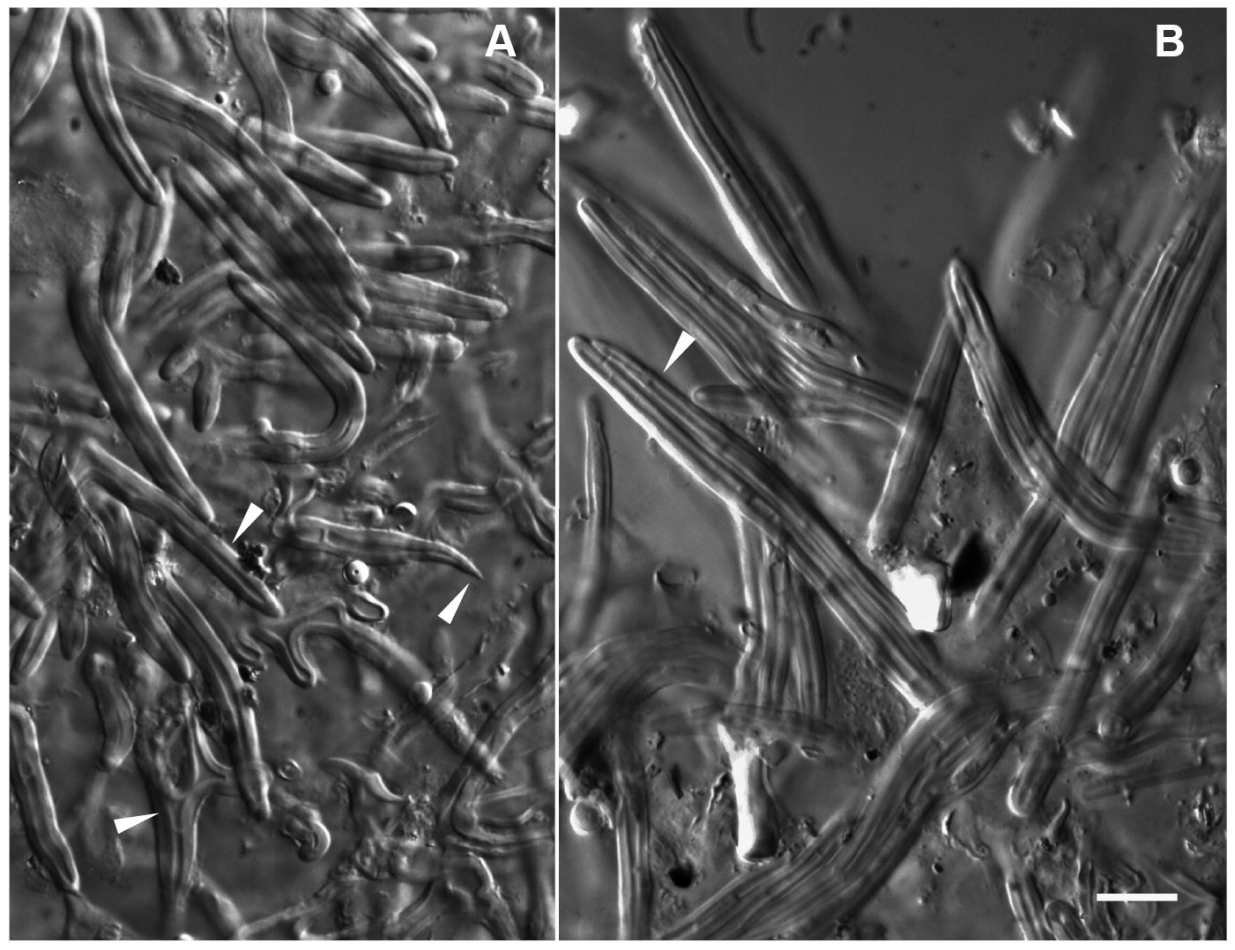
*Sebacina* cf. *guayanensis* + *Pakaraimaea dipterocarpacea* ssp. *nitida*. – A. Thick-walled hyphal net in outer mantle layer with thick walled, awl-shaped cystidia (arrow-head), aculeate distal hyphal ends (arrow-head), triangular inflations at ramification (arrow-head). – B. Cystidia glued together (arrow-head). Bar  =  10 µm. (Fig. From specimen 5MM2).

*Observations*: Specimen PD51 ITS1, 5.8S, ITS2 and 5MM2 5.8S, ITS2 were 99% similar to BM03M3 (504 from 506 nucleotides identical, and 336/338, respectively) and BM07M6 and 5MM2 LSU was 100% (464/464) and 99% (358/359) similar to BM03M3, respectively. The morphology and general anatomical features of specimen PD51, 5MM2 and BM07M6 were consistent with holotype specimen BM03M3. Characteristic features included the yellowish colour and woolly surface of ECM, the abundant smooth, thick-walled emanating hyphae and type B rhizomorphs with thick-walled hyphae, the transition between a dense plectenchyma and a pseudoparenchyma of thick-walled hyphae with a matrix in outer mantle layer, covered by a hyphal net of thick-walled hyphae with triangular inflations at ramifications, short protrusions and sometimes aculeate distal ends, frequent polytomies with three branches in the net, the yellow colour of cell walls, the clampless hyphae, the dense plectenchyma of thin-walled hyphae in deeper and inner layers. The main general anatomical difference between further studied material and BM03M3 were the frequent thick-walled, awl-shaped cystidia (50–90 µm long and 3.3–4.3 in diameter at proximal end) often glued together in 5MM2 (Fig. 3). Table 2 shows hyphal measurements of specimen BM03M3 and additional three similar specimens. Hyphal sizes and wall thickness in BM07M6 mantle and emanating elements were similar to BM03M3. The hyphal diameter and walls were particularly thick in 5MM2 (particularly so in the hyphal net) in comparison with BM03M3 and the remaining morphotypes. The hyphal diameter and walls were slightly thicker in PD51 than in BM03M3. Only hyphal measurements of specimen BM03M3 and BM07M6 are reported in the *S. guayanensis* description. An anatomotype identical to *S. guayanensis* was also observed on *Aldina* sp. roots (identified from morphology).

**Table 2:** Hyphal measurements of four ectomycorrhizal specimens belonging to *Sebacina guayanensis* from a *Pakaraimaea dipterocarpacea* ssp. *nitida* dominated forest.

	Hyphae	BM03M3	BM07M6	PD51	5MM2
Hyphal net	Diameter	2.3-2.7-3.5	2.5-3.3-3.7	2.5-3.5-4.0	3.0-4.0-5.0
	Wall thickness	0.5-1.0-1.7	1.0-1.2-1.3	1.0-1.5-1.7	1.3-1.5-2.3
Deeper layers	Diameter	1.7-2.5-3.0	2.7-3.0-3.5	2.5-3.3-3.3	2.3-2.7-3.5
	Wall thickness	Thin	Thin	Thin	Thin
Rhizomorph	Diameter	2.5-3.0-3.5	2.0-2.5-3.0	2.5-3.3-3.5	2.5-3.5-4.3
	Wall thickness	0.7-1.0-1.5	0.5-0.7-1.0	0.5-1.0-1.5	1.0-1.3-1.7
Emanating hyphae or cystidia	Diameter	2.7-3.0-3.3	2.3-3.0-3.3	3.0-3.3-4.3	3.0-3.5-4.0
	Wall thickness	1-1.1-1.5	0.5-1.0-1.3	1.0-1.1-1.7	1.0-1.3-1.7

Average of 10 measurements and minimum and maximum values.

#### *Sebacina tomentosa* B. Moyersoen & M. Weiß;, sp. nov. MycoBank MB808461; Index Fungorum IF550252 (Fig. 4; ref. [5], Figs. 9–10)

*Typus*: Venezuela, 4°20′N, 61°48′W, near Icabarú, in the Caroní headwaters, Estado Bolivar, leg. B. Moyersoen, 20 July 2007, ECM collected by root tracing from a *P. dipterocarpacea* Maguire & Ashton ssp. *nitida* Maguire & Steyerm. adult tree, specimen stored in CTAB, B. Moyersoen PD10, in LG.

**Figure 4:**
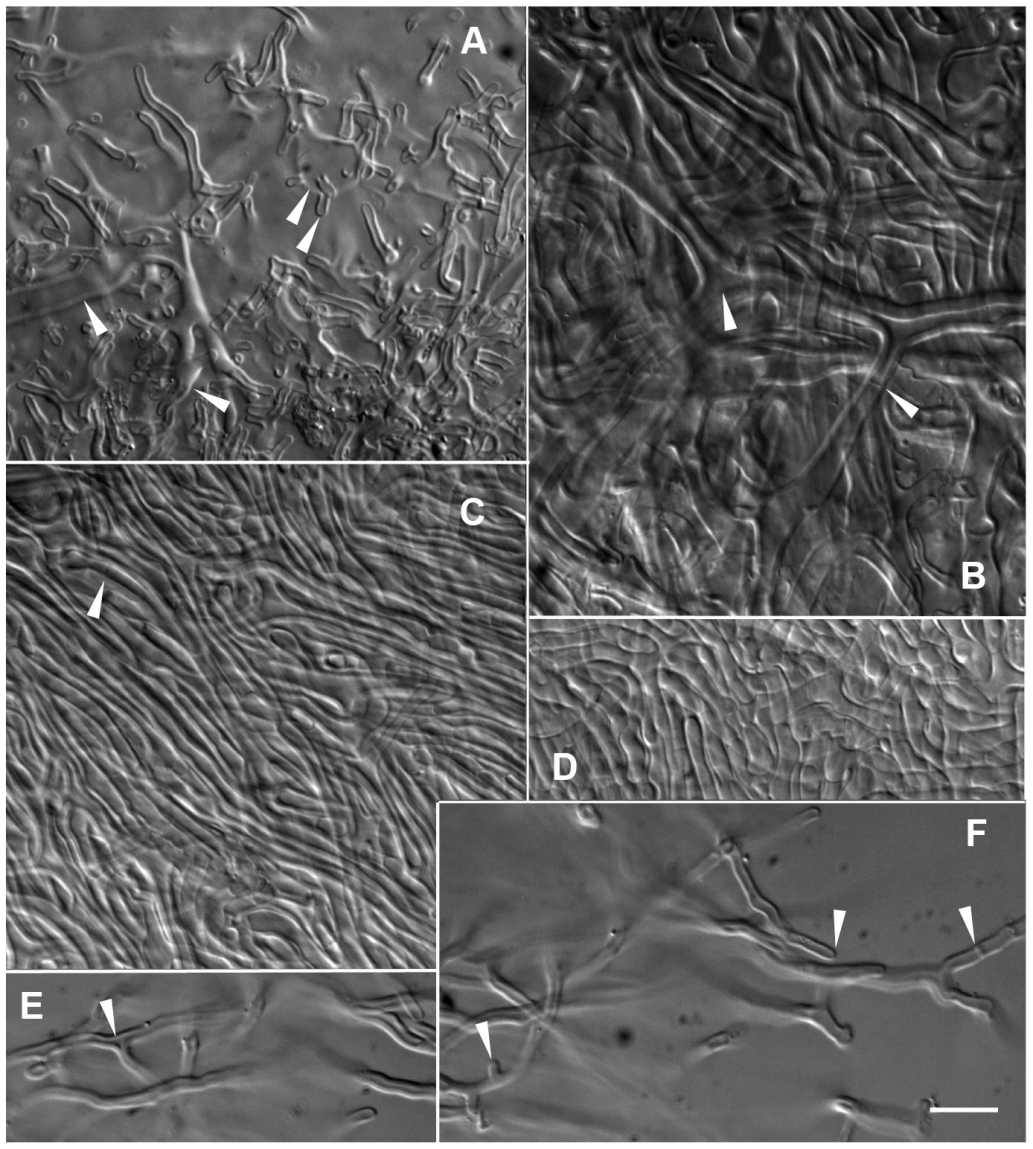
*Sebacina tomentosa* + *Pakaraimaea dipterocarpacea* ssp. *nitida*. – A–B. Plan views of outer layer. – A. Plan view of outermost mantle layer, loose net of hyphae of variable diameter (arrow-head), inflations (arrow-head), staghorn-like hyphae (arrow-head) with many finger-like outgrowths (arrow-head), septa infrequent, clampless. – B. Network of squarrosely branched hyphae, walls irregularly thick, triangular inflations at ramifications (arrow-head), septa infrequent, clampless (arrow-head). – C. Plan view of middle mantle layer, dense plectenchyma of thinner-walled, ramified hyphae, with a matrix (arrow-head). – D. Plan view of inner mantle layer, dense plectenchyma of ramified hyphae. – E–F. Emanating hyphae. Y-shaped ramification with triangular inflation (arrow-head), thick cell walls (E). Simple distal end (arrow-head), short protrusion (arrow-head), septa without clamps (arrow-head) (F). Bar  =  10 µm. (Fig. from specimen PD10).

*Short description*: The smooth to short spined or cottony ECM of *S. tomentosa* are characterized anatomically by the thick-walled staghorn-like hyphae in the outermost mantle layer. The outer ectomycorrhizal mantle layer is a loose plectenchyma of squarrosely branched hyphae of variable (2-3.7-6.5 µm) diameter, becoming more densely arranged in deeper layers. A matrix is present in the deeper layers. Cell walls, which are colourless, are thick or thin in the outer mantle layer, and thinner in the deeper and inner layers. Polytomies with three or more branches are present in the outer mantle layer. Emanating hyphae are thick-walled and 2-2.3-2.5 µm diam. Nuclear rDNA data clearly support the separation of this species from other known species in the group.

*Etymology*: Epithet *tomentosa* relating to the short-spined surface due to the abundant staghorn-like hyphae in ectomycorrhizal outer layer.

*Description of the species*: *Ectomycorrhizal systems* with a smooth to short-spined or cottony surface; emanating hyphae occurring in patches; rhizomorphs lacking; mycorrhizas belonging to the short distance *exploration type*. *Outer ectomycorrhizal mantle layer*, a loose plectenchymatous network of squarrosely branched hyphae (hyphae multiply ramified with short, frequent Y-shaped or almost rectangular branches, type E in ref. [50]) of variable diameter (2-3.7-6.5 µm) becoming more densely arranged in deeper layers; cell walls smooth, colourless, either irregularly thick (0.5-1.3-2.5 µm) or thin; staghorn-like hyphae similar to *cystidia* with many finger-like outgrowths frequent in the outermost layer; polytomies with three or more branches; hyphal inflations including triangular inflations at ramifications; short hyphal protrusions present in the outer layer. *Deeper and inner mantle layers*, a dense plectenchymatous network of ramified, cylindrical hyphae, with a matrix; cell walls smooth, colourless, generally thin. *Emanating hyphae* smooth, 2-2.3-2.5 µm diam.; cell walls smooth, colourless, 0.5-0.7-1 µm thick, even in thickness or thinner at distal end; distal end simple or with a protuberance; ramifications Y-shaped or branching at 90°, often with triangular inflation; short protrusions occurring throughout. *Anastomoses* short, open. *Clamps* absent, septa infrequent, straight or curved. *Mantle preparation*, colour reaction test according to Melzer not performed.

*Material studied*: Venezuela, 4°20′N, 61°48′W, near Icabarú, in the Caroní headwaters, Estado Bolivar, leg. B. Moyersoen, 20 July 2007, ECM collected by root tracing from a *P. dipterocarpacea* Maguire & Ashton ssp. *nitida* Maguire & Steyerm. adult tree, specimen stored in CTAB, holotype designated as B. Moyersoen PD10 in LG. Further material studied: leg. B. Moyersoen, 17 July 2007, specimen in CTAB in Herb. B. Moyersoen PD91; leg. B. Moyersoen, 24 July 2008, specimen in CTAB in Herb. B. Moyersoen 1AM1; leg. B. Moyersoen, 31 July 2008, specimen in CTAB in Herb. B. Moyersoen 10PDM3.

*Molecular characters*: The identical sequence from three strains (PD10, PD91, 10PDM3) of *S. tomentosa* sequenced in this study is given in the following format: [3′ 18S]ITS1*5.8S*ITS2[5′ 28S].

[CATTA]TTGATTACGAATTGTTGCTGTCTGTGCTGGCTCCGGCAAGTGCACGTCGGTGACTTTCATCCAACACCCCTGTGCACTCTTGGCCTCTTGCTAGCTTCGGCTGGCTGAGGATTTTTTACACACTCACTCGAATGTAATGAAAAACTATGGTTGTGCGCAAGCACGAATGTAC*AACTTTCAACAACGGATCTCTTGGCTCTCGCATCGATGAAGAACGCAGCGAAATGCGATAAGTAATGTGAATTGCAGAATTCAGTGAATCATCGAATCTTTGAACGCACCTTGCACCCTTTGGCATTCTGAAGGGTATGCTCGTTTGAGTGTCAT*TGTACTCTCATACTCTCTGATCCTTTTGGATTTGGGAGCGATGGACTTGGGTGTTGCTGCTTTACCGTGGCTCACCTGGAATGCATTAGTGCATCTCTTGGTTGGGCATAGTACGGCGTGATAAAGTGTCTTCGCCGGCACCTCGCAAGAGGGTGGCTGACCAGGAGCTCTGTGCTTCCTAATGGTCTTCGGACAATCTCTGACGGCTTGACCTCAAAT[CGAGTAGGACT]

*Observations*: Specimens PD91 ITS1, 5.8S, ITS2 and 10PDM3 5.8S, ITS2 were 100% identical (520 from 520 nucleotides identical, and 258/258, respectively) to PD10. No ITS sequence could be retrieved from 1AM1 ECM associated with *Aldina* sp., but the LSU was similar to PD10 (355/357, 99% similarity). PD91, 10PDM3 and 1AM1 morphology and anatomy were generally consistent with PD10. The characteristic features of the ECM included: the smooth or short-spiny surface of the ECM with emanating hyphae in patches, absence of rhizomorphs, loose network of squarrosely branched hyphae with variable diameter including staghorn-like hyphae with many finger-like outgrowths similar to cystidia in outer mantle layer, colourless, smooth walls, which were thick or thin in outer mantle layers and thinner in deeper and inner layers, polytomies with three or more ramifications in mantle outer layer, inflations including triangular inflations at ramifications, hyphal protuberances in outer mantle layer, clampless hyphae, matrix in deeper or inner layers, smooth, thick-walled emanating hyphae, dense plectenchyma of mostly cylindrical hyphae in deeper and inner layers. Septa in outer mantle layer were more frequent in 10PDM3 than in remaining specimens and no matrix was observed in 1AM1.

#### Sebacinales specimen 6MM2 (Fig. 5; ref. [5], Fig. 11)

*Description of the species*: The morphology of e*ctomycorrhizal systems* was not recorded. Based on the anatomy of emanating elements, belonging to a transition between the short distance and the smooth subtype of medium distance *exploration type*. *Outer ectomycorrhizal mantle layer*, a loose network of squarrosely branched hyphae (type E in ref. [50]), becoming densely arranged in deeper layers and giving rise to abundant emanating hyphae; hyphae mostly cylindrical, 2-3-3.7 µm diam., with frequent irregular inflations and short protrusions; ramifications often with triangular inflations; polytomies with three branches; frequent clusters of hyphae emanating from the outer layer; walls smooth, colourless, irregularly thick (0.5-1-1.5 µm). *Middle mantle layers* a dense plectenchyma of ramified, cylindrical hyphae; walls colourless, smooth, generally thin. *Inner layers*, a transition between a plectenchyma and a pseudoparenchyma; walls smooth, colourless, thin. *Rhizomorphs* frequent, uniform compact, 10–50 µm diam.; hyphae 2.5-2.7-3.3 µm diam.; walls colourless, smooth, 0.5-0.7-1 µm thick, thinner at hyphal distal end. *Emanating hyphae* abundant, smooth, 2.5-2.7-3 µm diam., with simple distal end; walls smooth, colourless, 0.7–1 µm thick, thinner at hyphal distal end; ramifications Y-shaped or branching at 90°, often with triangular inflation; short protrusions at proximal end. Some emanating hyphae similar to awl-shaped *cystidia* with basal dichotomous or trichotomous ramifications. *Anastomoses* short, open. *Clamps* absent, septa often curved. *Mantle preparation*, colour reaction test according to Melzer not performed.

**Figure 5:**
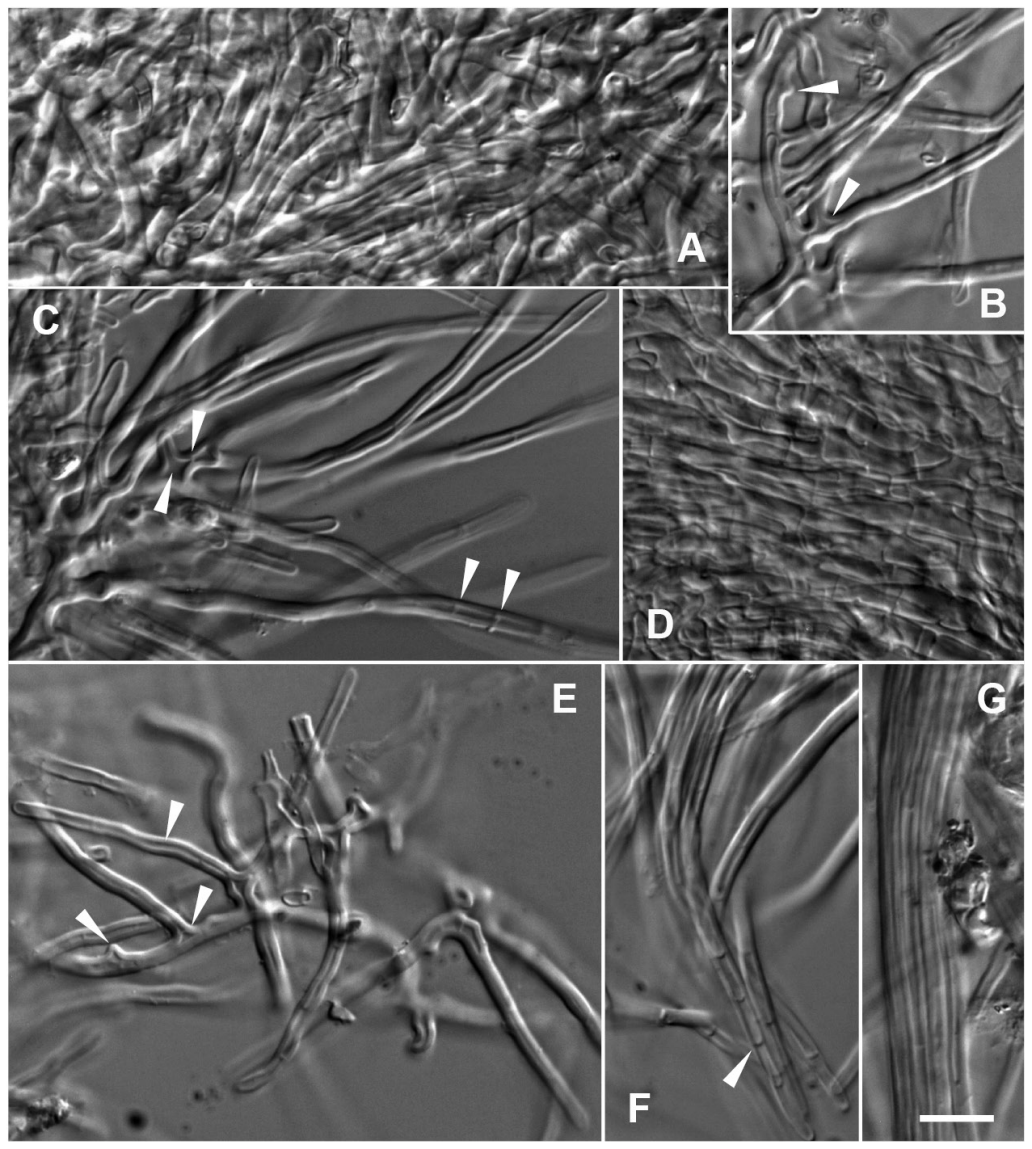
Sebacinales specimen 6MM2 + *Pakaraimaea dipterocarpacea* ssp. *nitida*. – A–C. Plan views of outer layer, loose network of squarrosely branched, thick-walled hyphae giving rise to abundant emanating hyphae (A, B, C), triangular inflation at ramifications (arrow-heads) (B, C), short protrusions (arrow-head) (B), polytomy with three branches (arrow-head) (C), septa frequent, without clamps (arrow-heads) (C). – D. Plan view of middle mantle layer, dense plectenchyma of ramified hyphae, cell walls generally thinner than in outer layer. – E–F. Emanating hyphae, thick cell walls, Y-shaped ramification, triangular inflation at ramification (arrow-head), short protrusion (arrow-head), shorter emanating hyphae similar to awl-shaped cystidia (arrow-head) (E). Clampless septa frequent, often curved (arrow-head) (F). – G. Rhizomorph, thick walled hyphae of uniform diameter running in parallel. Bar  =  10 µm.

*Material studied*: Leg. B. Moyersoen, 31 July 2008, DNA extract in Herb. B. Moyersoen 6MM2.

*Molecular characters*: The ITS sequence from Sebacinales 6MM2 sequenced in this study is given in the following format: 5.8S (partial sequence)*ITS2[5′ 28S].

GAGTGTCATTGTAC*TCTCACGCTCTCTAATTCGGATTCGCGTTCGGATTGGGAGTGGTGGACTTGGGTGTTGCCGCTTCGCTGTGGCTCACCTCAAATGTGTTAGTGCAACTGTTGATTAGACATAGTACGGCGTGATAAGTGTCTTCGCCGGCACCTCACAAGAGGGTGGCTAATCGGGAGCTCTGTGCTTCAAACTGTCTCTGGACAATCTCTGACAACTTGACCTCAAAT[CGAGTAGGACT]

### Distribution and frequency of *Sebacina* ECM in *Pakaraimaea dipterocarpacea* forest

Sebacinales is a frequent fungal group in the plot; *Sebacina guayanensis* and *S. tomentosa* were particularly well represented. Amongst the total of 97 sequences obtained from 113 DNA extracts, 11 sequences (11%) belonged to the three *Sebacina* species. Most sequences were from *S*. *guayanensis* (four sequences) and *S. tomentosa* (six sequences). DNA was extracted from a total of 64 root systems from the nine soil cores scattered in the plot, and 56 fungal DNA sequences were obtained. The three *Sebacina* species were recorded from five sequences and they were present in five out of nine (55%) soil cores. *S. tomentosa* was represented by three sequences from two soil cores, *S. guayanensis* by two samples (including an unsequenced morphotype) from two soil cores. Sebacinales specimen 6MM2 was a singleton.

### ITS and LSU comparisons of *Sebacina guayanensis*, *S. tomentosa* and Sebacinales specimen 6MM2 with sequences obtained from GenBank and UNITE

None of the ITS and LSU sequences of Sebacinales species associated with *P. dipterocarpacea* matched identified species in a BLASTn search against the GenBank nucleotide database performed on 27 September 2013. A significant match with undescribed species was found between the ITS sequences of *S. guayanensis* and an uncultured Sebacinales voucher ECM17 (JN168754) (497 from 514 nucleotides identical, 97%), and of Sebacinales specimen 6MM2 and an uncultured Sebacinales voucher ECM84 (JN168756) (251/258, 97%), respectively. Both hitherto undescribed ECM were associated with *Dicymbe corymbosa* in the Upper Potaro River Basin in the Pakaraima Mountains of Guyana [36].

### Molecular phylogenetic placement of *Sebacina guayanensis*, *S. tomentosa* and Sebacinales specimen 6MM2

**Figure 6:**
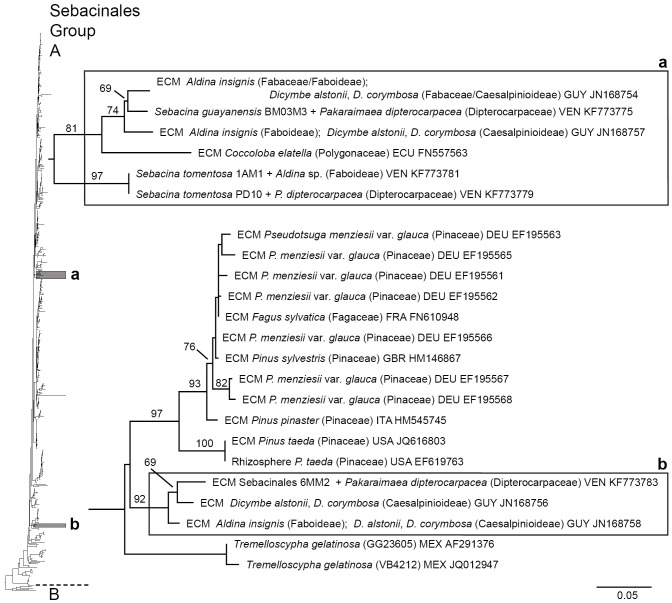
Phylogenetic placement of three Sebacinales species from a *Pakaraimaea dipterocapacea* forest. Best ML tree based on combined analysis of ITS and LSU sequences, found by heuristic search in RAxML [48] using the GTRCAT model of DNA substitution and 1000 replicates of integrated rapid bootstrapping. The tree was rooted with four *Sebacina vermifera* strains (AY505548, DQ983816, DQ520096, AY505555) and *Piriformospora indica* (AY505557) strain belonging to Sebacinales Group B. Bootstrap values < 50% are not shown. Genetic distances are given in terms of expected number of substitution events per nucleotide (see bar). GUY: Guyana, VEN: Venezuela, ECU: Ecuador, DEU: Germany, FRA: France, GBR: Great Britain, ITA: Italy, USA: United States of America, MEX: Mexico.

**Figure 7:**
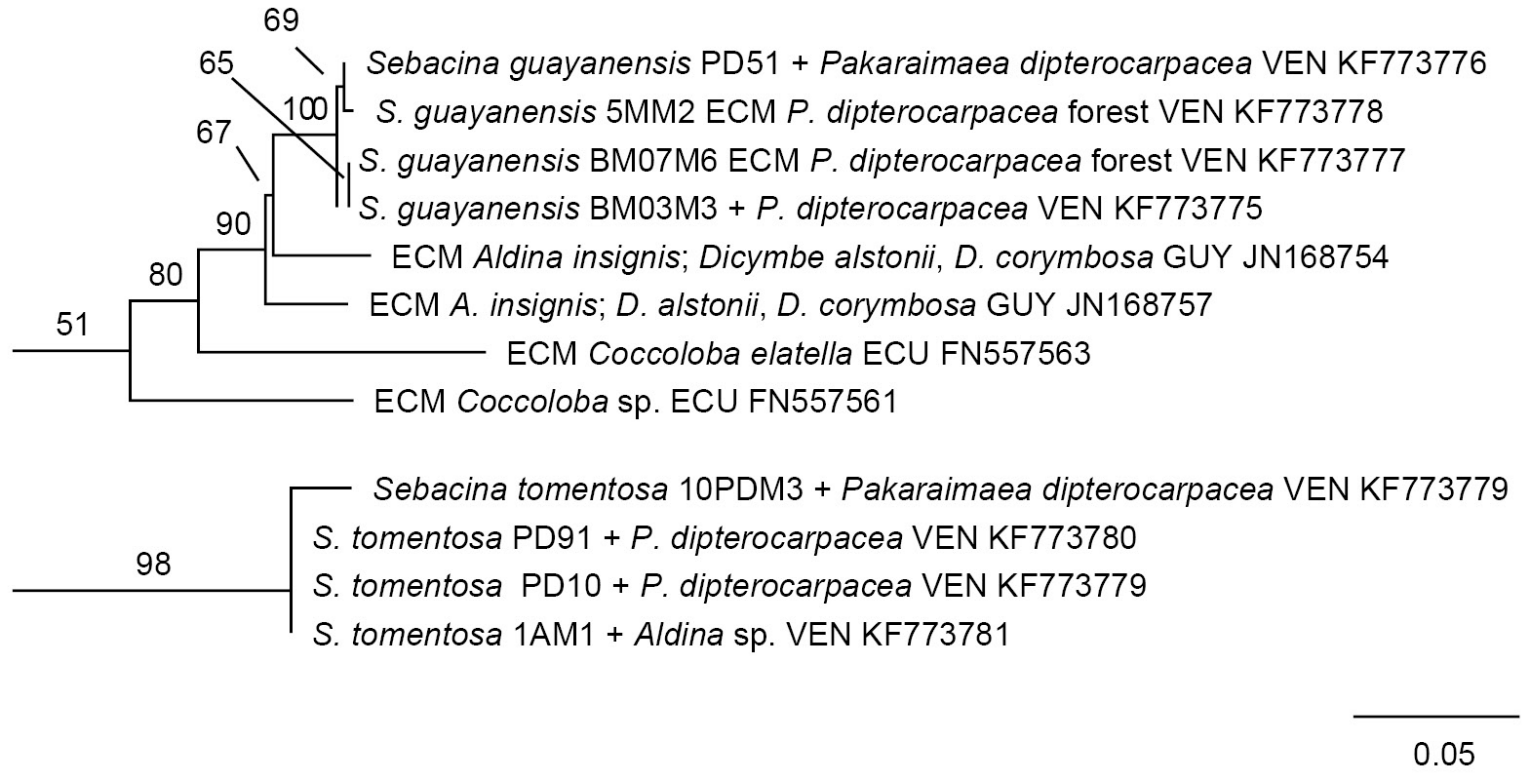
Phylogenetic placement of specimens belonging to *Sebacina guayanensis* and *S. tomentosa* from a *Pakaraimaea dipterocapacea* forest. Portion of best ML tree based on combined analysis of ITS and LSU sequences, resulting from heuristic search in RAxML [48] with 1000 replicates of integrated rapid bootstrapping and using the GTRCAT model of DNA substitution. The branch length leading to *S. guayanensis* specimens BM03M3 and BM07M6 is almost nil; this clade has been transposed horizontally for topological clarity. Bootstrap values < 50% are not shown. Genetic distances are given in terms of expected number of substitution events per nucleotide (see bar). VEN: Venezuela, GUY: Guyana, ECU: Ecuador.

The preliminary phylogenetic analysis of data set A using the complete data set analysed in ref. [4] as profile alignment demonstrated that *S. guayanensis*, *S. tomentosa* and Sebacinales 6MM2 belonged to Sebacinales Group A (data not shown), each in a different clade. Of these, Sebacinales specimen 6MM2 obtained the most basal position in Group A (Fig. 6). None of these three clades included identified species. The tree topology suggested a sister relationship between the clades including *S. guayanensis* and *S. tomentosa* and between Sebacinales specimen 6MM2 and *Tremelloscypha gelatinosa* strains from Mexico, but the nodes were not statistically significant (6% and 26% bootstrap value, respectively). *Sebacina guayanensis* and *S. tomentosa* appeared as well-supported phylogenetic units in our RAxML analysis of data set B (Fig. 7).

**Figure 8:**
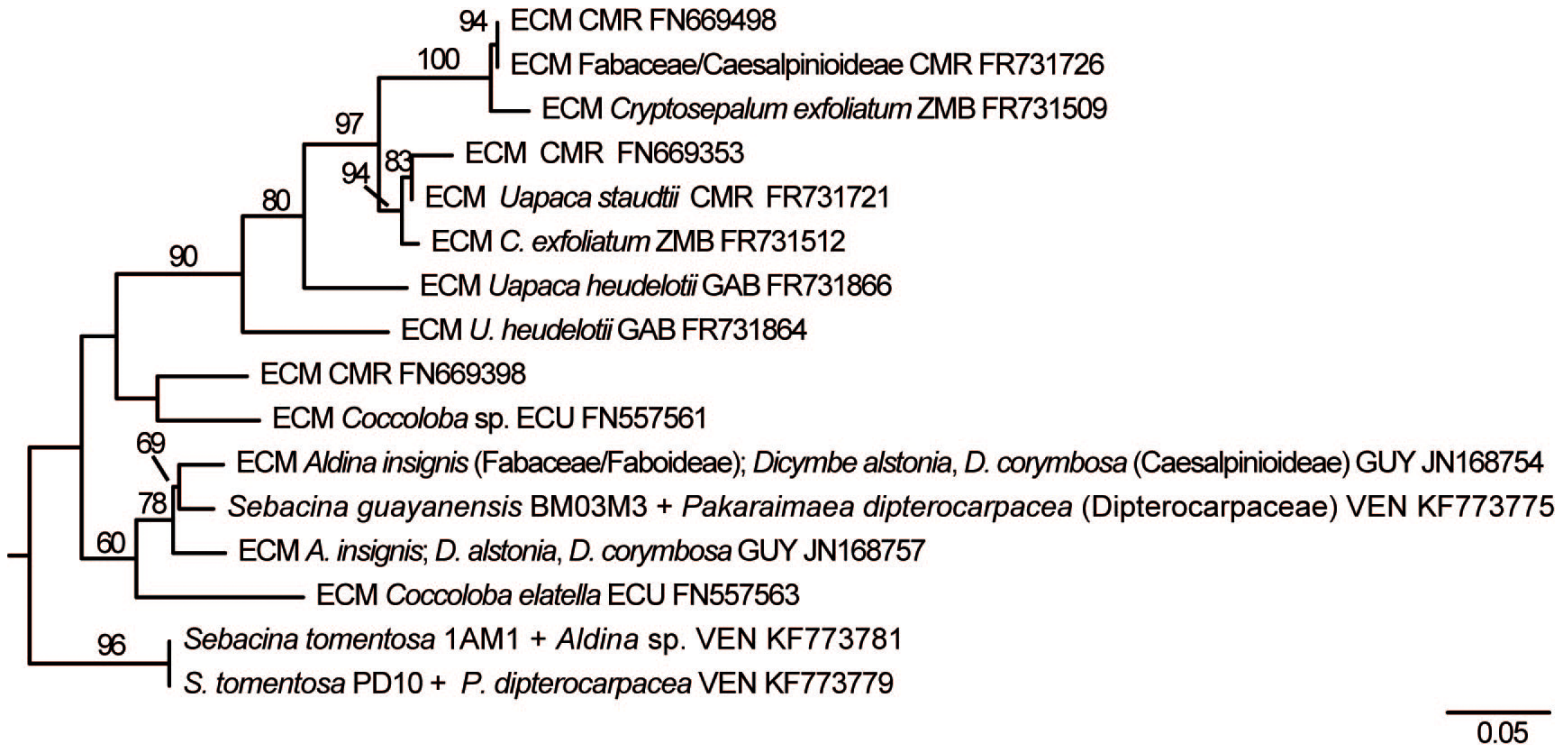
Phylogenetic placement of two Sebacinales species from a *Pakaraimaea dipterocapacea* forest, before pruning of sequences FN669398 and FN557561. Portion of best ML tree based on combined analysis of ITS and LSU sequences, resulting from heuristic search in RAxML [48] with 1000 replicates of integrated rapid bootstrapping and using the GTRCAT model of DNA substitution. Bootstrap values < 50% are not shown. Genetic distances are given in terms of expected number of substitution events per nucleotide (see bar). CMR: Cameroon, ZMB: Zambia, GAB: Gabon, ECU: Ecuador, GUY: Guyana, VEN: Venezuela.

Each of the three clades with *Sebacina* species from *P. dipterocarpacea* ECM also included strains associated with different tree host families including the Fabaceae (Faboideae and Caesalpinioideae/Amherstieae) and the Polygonaceae that were present or not in the studied forest (Figs. 6, 7). A regional pattern could be observed amongst Sebacinales strains phylogenetically related with *S*. *guayanensis* and Sebacinales specimen 6MM2. Specimen 6MM2 was related with strains (JN168756, JN168758) collected in a forest dominated by the leguminous host *Dicymbe corymbosa* situated ca 240 km apart in the same Guayana region. *Sebacina guayanensis* clustered with strains (JN168754, JN168757) collected in eastern areas, in Guyana (in a *D. corymbosa* forest) and in western areas, in an Ecuadorian lowland rain forest (strain FN557563 in association with a *Coccoloba* species). The tree topology resulting from the RAxML analysis of data set A suggested a relationship between *S*. *guayanensis* clade and two clusters including one well supported clade with paleotropical strains collected in Africa, but this relationship was not statistically significant (8% bootstrap value; Fig. 8). This relationship was not apparent in the tree resulting from RAxML analysis of the complete data set (data set B; result not shown).

### Molecular phylogenetic relationships between Sebacinales species associated with *P. dipterocarpacea* and previously described Sebacinales ECM morpho-/anatomotypes

**Figure 9:**
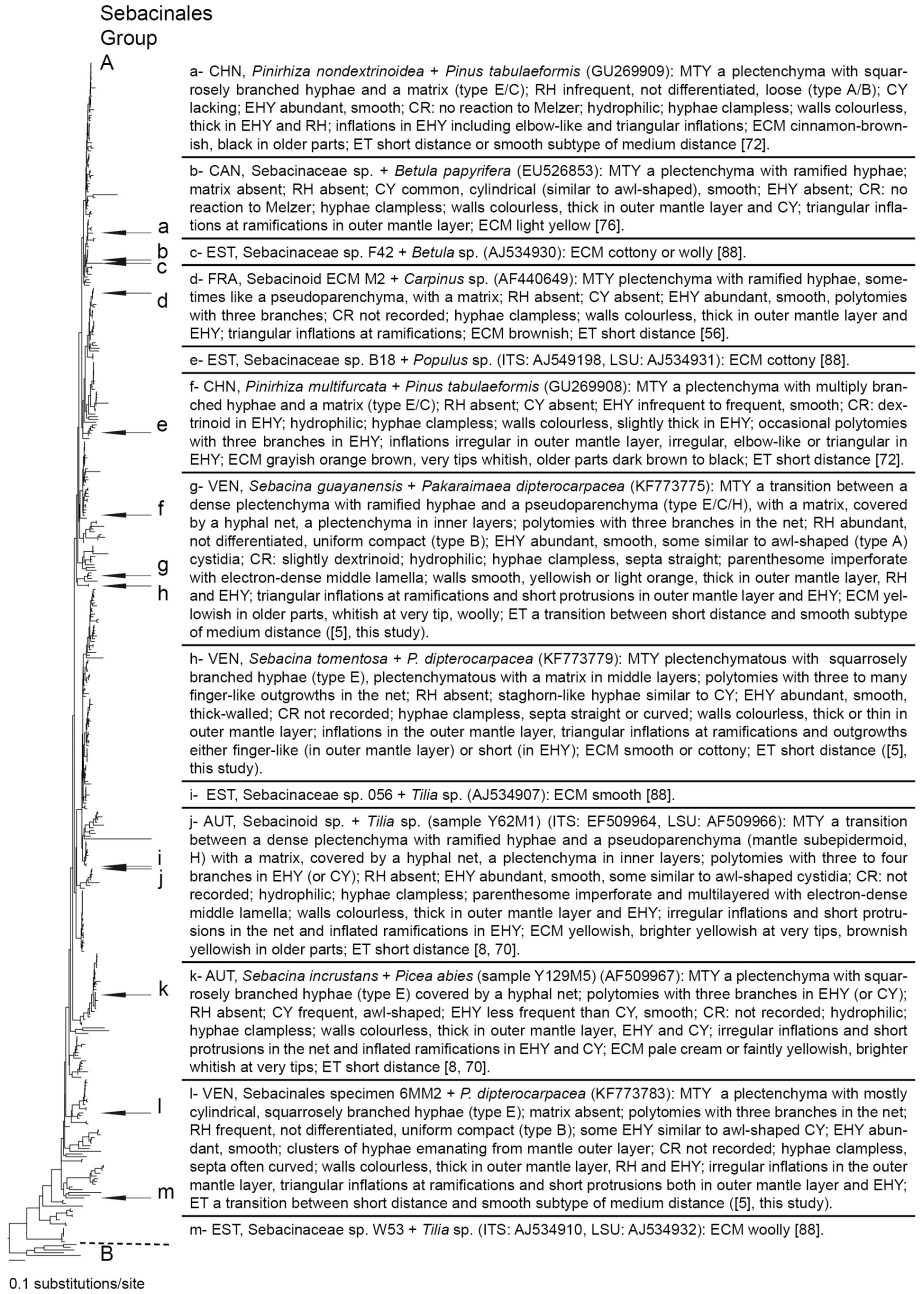
Phylogenetic placement of described Sebacinales ECM morpho-/anatomotypes worldwide. Best ML tree based on combined analysis of ITS and LSU sequences, resulting from heuristic search in RAxML [48] with 1000 replicates of integrated rapid bootstrapping using the GTRCAT model of DNA substitution. The tree was rooted with four *Sebacina vermifera* strains (AY505548, DQ983816, DQ520096, AY505555) and *Piriformospora indica* (AY505557) strain belonging to Sebacinales Group B. Genetic distances are given in terms of expected number of substitution events per nucleotide (see bar). Sebacinales morphotype *Quercirhiza dendrohyphidiomorpha* + *Quercus suber* L. [71] is missing for the lack of available DNA sequences. MTY: mantle type; RH: rhizomorph; CY: cystidia; EHY: emanating hyphae; CR: color reaction; ET: exploration type. Mantle and cystidia types according to ref. [50]. Exploration types according to ref. [86]. Habit of Sebacinaceae sp. F42 + *Betula* sp., Sebacinaceae sp. B18 + *Populus* sp., Sebacinaceae sp. 056 + *Tilia* sp. and Sebacinaceae sp. W53 + *Tilia* sp. defined from plan views in ref. [88]. CHN: China, CAN: Canada, EST: Estonia, FRA: France, VEN: Venezuela, AUT: Austria.

A total of 13 sequenced Sebacinales ECM morpho-/anatomotypes (including this study) have been described to date. A preliminary RAxML analysis including the complete data set used in ref. [4] as profile alignment indicated that all described Sebacinales ECM anatomotypes belong to the Group A (data not shown); the sequences were distributed across the Sebacinales Group A phylogenetic tree (Fig. 9). None of the Sebacinales species associated with *P. dipterocarpacea* clustered with previously described morpho-/anatomotypes.

## Discussion

### Biogeography of Sebacinales associated with *Pakaraimaea dipterocarpacea*

This is the first molecular phylogenetic study with an emphasis on tropical ECM Sebacinales. In contrast with the view that there is no clear biogeographic pattern in Sebacinales clades [1], [3], [4], [51], [52]; but see ref. [53]), the present study suggests that tropical ECM trees including *P. dipterocarpacea* can host typically tropical Sebacinales species (but see ref. [24]) with no close relatives in temperate areas. The additional observation of an African-only clade indicates that Sebacinales Group A strains, with no closely related temperate species, may have diversified both in the neo- and the paleotropics. The biogeographic pattern in Sebacinales observed in former phylogenetic studies has been attributed both to an efficient dispersal [1] and the lack of specificity and co-evolutionary processes [7]. It has been suggested [53] that Sebacinales inferences based on LSU are conservative and may not be appropriate for estimations of distribution ranges.

We have used a similar strategy of phylogenetic analyses as reported in ref. [4], using concatenated ITS and LSU sequences. The observation of tropical-only clades for the first time in the present study may be attributable to a greater sampling of tropical ECM Sebacinales sequences in our datasets. In addition to Sebacinales strains associated with *Coccoloba* sp. in Ecuador [33] and *P. dipterocarpacea* in Venezuela [5], [11], [12], reported neotropical lowland ECM Sebacinales strains are from a *Dicymbe* forest in Guyana [36]. The biogeography of *Dicymbe* ECM Sebacinales phylogenetically distant to the species associated with *P. dipterocarpacea* is unresolved (data not shown). Further records are necessary to confirm the extent of neotropical ECM Sebacinales regional patterns and to test whether *S. tomentosa* belongs to an additional tropical-only clade. Our results (see Fig. 8) also suggest that there is a possible phylogenetic relationship between neotropical ECM Sebacinales strains including *S. guayanensis* and African strains, however, the bootstrap support for this was very low. Similarly, a possible African-neotropical link has been reported for a basal *Inocybe* clade belonging to subgenus *Inocybe* [54] including strains associated with *P. dipterocarpacea* [12]. To our knowledge, amongst the total of 15 reported Sebacinales strains collected in Dipterocarpaceae-dominated forests across the tropics ([11], [12], [29], [30], [37], [55], this study), Dipterocarpacean hosts were involved only in one instance (FR731519) (*Monotes* sp.) in Africa [37] and three instances in the neotropics ([5], [11], [12], this study). More sequence data are necessary to confirm this neotropical-paleotropical link and to test whether Dipterocarpaceae and possibly other tropical tree families with relatives both in Africa and South America such as the Caesalpinioideae (Amherstieae) contributed to this hypothetical migration route.

This study is consistent with the general knowledge that Sebacinales include many host generalist species (e.g. [1], [6], [56]). There is evidence from molecular studies in temperate areas that identical Sebacinales Group A species may form ECM with different tree host genera [7], [56]; identical species may also form both ECM and arbutoid or pyroloid ectendomycorrhizas [1], [4], [51] or mycorrhizas with heterotrophic or partly heterotrophic orchids [1], [2], [57]. Recent phylogenetic studies have already suggested that endophytic Group A Sebacinales are involved in ECM associations [7]. ITS- or LSU-identical ECM Sebacinales strains were recovered from different native tree families or species both in the paleo- [37], and the neotropics [12], [28], [36], respectively. Recent results [34] suggested that native ECM fungi including Sebacinales can jump from African ECM Fabaceae to eucalypts in mixed forest plantations. Sebacinales strains were recovered from ECM roots of the otherwise ECM specific tree host *Alnus acuminata* in Mexico [35]; our phylogenetic analysis showed that two of these strains (HQ271417, HQ271373) were closely related (bootstrap support of 100%) with a Sebacinales strain (FJ196961) associated with oak in the same area. The association of *S. tomentosa* with an *Aldina* species indicates that it is not specific to *P. dipterocarpacea*. Our study also showed that a Sebacinales strain (FN557563), presumably specific to *Coccoloba* in an Ecuadorian forest [33], was phylogenetically closely related with *S. guayanensis* and strains associated with Fabaceae (JN168754, JN168757) in Guayana (Fig. 6). The present study confirms that identical or phylogenetically close Sebacinales strains are associated with *P. dipterocarpacea* and other plant host families either occurring in the same forest or within the same region. These results are consistent with a regional pattern indicating that *P. dipterocarpacea* ECM fungal community includes a great proportion of host generalists [11], [12], [24].

Both the regional pattern observed in Sebacinales clades and the association of phylogenetically related strains with host plants belonging to phylogenetically distant families suggests that host jump and possibly dispersion are important radiation mechanisms for this fungal group in neotropical lowland forests. ECM tree species in neotropical rain forests are usually scattered in a wide range of vegetation types where most tree species are arbuscular mycorrhizal (AM) [12]. They also form monodominant or codominant stands of ECM trees surrounded by a matrix of mostly AM tree species (e.g. [11], [58], [59]). Whether these neotropical lowland forest stands with a great component of ECM trees are remnants of more extensive ECM forests in the past is not known. The extent of the regional pattern observed in two Sebacinales clades in this study is consistent with a high dispersal capacity of this fungal group [1]. Precise mechanisms by which Sebacinales propagate still have to be investigated. Gelm et al. [60] suggested that a great proportion of ECM fungi including Sebacinales colonized Svalbard by long-distance dispersal. Spore dispersal by wind has been proposed for fungi producing aerial spores such as the Inocybaceae [54], [60] and *Pisolithus* [61]. Kropp [62] suggested that mycophagy by animals might be a means of spore dispersal for ECM fungi in neotropical lowland forests. During the field trip in June 2007, it was possible to observe deer (presumably a *Mazama* species) feeding on *Cantharellus guianensis* fruitbodies (identical to specimen BM07C6 in ref. [12]). The importance of animals including mammals in ECM spore dispersal in lowland neotropical forests deserves further investigation. Spore dispersal is possibly not the main way of propagation in Sebacinales. Members of Sebacinales Group B can produce chlamydospores in the soil [63]. Based on a phylogenetic study it has been suggested that fungi from Sebacinales Group B co-migrated with Vaccinioideae from North America to the Andes before a land bridge was established between the two continents [53]. This migration route might have involved stepping stone dispersal between islands. The hypothesized capability of Sebacinales strains to connect with different host species (e.g. [4], [51], [56], [57]) or to become endophytic if no mycorrhizal partner is available [1] might contribute to efficient host jumps. It has also been suggested that root-endophytic fungi might be seed-dispersed during their life-cycle [64]. The capacity of Sebacinales to become systemic endophytes and to be dispersed by plant seeds deserves further investigation. Dispersion and host jump have already been proposed as radiation mechanisms for phylogenetically related neotropical *Inocybe* species associated with *P. dipterocarpacea* and Fabaceae [12]. *Coccoloba* is a diverse [65] dual ECM/AM host genus [66], [67]. This neotropical genus [65], which occurs usually scattered and occupies a wide range of ecological niches in the Guayana region [12], may act as a nurse host and be an important element of the ECM plant community favoring the radiation of probably efficiently dispersing fungal groups such as the Sebacinales. Interestingly, Sebacinales associated with *C. elatella* in Ecuador appeared basal in the phylogroup including *S. guayanensis*. It is possible that *Coccoloba* contributed to the migration of sebacinalean fungi from Western neotropical forests to the Guayana region. More intensive surveys of ECM fungi on *Coccoloba* are needed to test the relevance of this genus for the radiation of ECM fungi in neotropical lowland forests. The phylogeny of Sebacinales associated with *P. dipterocarpacea* further supports the hypothesis that host jump and dispersion are important radiation mechanisms of ECM fungi in neotropical lowland forests. These radiation mechanisms might contribute to the recently reported [68] great overall β diversity in soil fungi from Western Amazonian forests.

In contrast to the original hypothesis that *P. dipterocarpacea* hosts ECM fungi including Sebacinales with relictual disjunct distribution [11], this study indicates that several colonization events may have been responsible for shaping the Sebacinales community on this endemic species. At least two Sebacinales species associated with *P. dipterocarpacea* (*S. guayanensis*, *S. tomentosa*) belong to more derived phylogroups (Fig. 6); a possible scenario for one of these species (*S. guayanensis*) is immigration from Western neotropical lowland forests with *Coccoloba* contributing to the radiation of this species. The clustering of the third species associated with *P. dipterocarpacea* in a relatively basal clade in Sebacinales Group A is consistent with an old origin of the association with *P. dipterocarpacea*. Association of strains within this clade with ECM Fabaceae including *Dicymbe* might be attributable both to the host generalist status of Sebacinales and to long term coexistence of these two ECM tree families within the Guayana region. More ITS and LSU sequences are needed to test the molecular phylogenetic relationships between neotropical and paleotropical Sebacinales associated with Dipterocarpaceae. We frequently found Sebacinales on *P. dipterocarpacea* and it is surprising that no Sebacinales sequences were reported from a *P. dipterocarpaceae* ssp. *dipterocarpacea* forest in Guyana [24]. However, since the sampling strategy chosen in ref. [24] did not allow to get a complete representation of the fungal community, Sebacinales records can be expected in further *P. dipterocarpaceae* ssp. *dipterocarpacea* ECM surveys [24]. Sebacinales reports are also still lacking for the second neotropical Dipterocarpacean genus, *Pseudomonotes*, which belongs to the Monotoideae. Sebacinales have probably been undersampled in African Monotoideae taking into account the frequent presence of this fungal group in other tree families (e.g., [32], [37], [69]) in Africa.

The present study corroborates the earlier conclusion that *P. dipterocarpacea* ssp. *nitida* ECM fungal community includes a conspicuous proportion of host generalists [11], [12]. These include ECM fungal lineages such as the Inocybaceae and Sebacinales, with no close temperate relatives known from molecular studies. This is consistent with the hypothesis of a long-term diversification of ECM fungi in tropical areas [11]. Weakly supported links between neo- and paleotropical strains have been suggested in the Inocybaceae [12], [54] and the Sebacinales (this study), but the data are still insufficient to test whether this possible pattern can be attributed to the hypothetical relictual disjunct distribution of ECM fungal clades that co-evolved with the Dipterocarpaceae.

### Structural diversity of Sebacinales ECM associated with *Pakaraimaea dipterocarpacea*

This study is the first combined anatomical and molecular phylogenetic study of tropical ECM Sebacinales. The results suggest that ECM anatomical characters can be used for the description of species within Sebacinales Group A. Combined molecular phylogenetic analysis and anatomical descriptions indicated that the three ECM anatomotypes associated with *P. dipterocarpacea* belong to three distinct species of Sebacinales. Two of these species are consequently described in the present study.

*Sebacina guayanensis*, *S. tomentosa* and the species including specimen 6MM2 share important taxonomic features with previously described ECM anatomotypes belonging to temperate Group A Sebacinales. A summary of important anatomical features of the three Sebacinales ECM taxa associated with *P. dipterocarpacea* together with a synthesis of descriptions available to date on sequenced Group A Sebacinales morphotypes worldwide is shown in Fig. 9. Sebacinales ECM micromorphology is characterized by a combination of ultrastructural [3], [70], and anatomical features [50]. An ultrastructural feature observed in Sebacinales ECM is the dolipore with imperforate, multilayered parenthesomes, and an electron-opaque middle lamella [56], [70]. Amongst the anatomical features, awl-shaped and dichotomously or multiply ramified cystidia, which have not been recorded from the hymenium in Sebacinales [3], were observed in Sebacinales ECM [50]. Features shared between the Sebacinales ECM associated with *P. dipterocarpacea* and published descriptions of temperate Sebacinales ECM have been discussed in ref. [5]. Dolipores with imperforate parenthosomes and an electron-opaque middle lamella were recorded in *S. guayanensis* ECM. Hyphae were clampless in the three species, as in all species of Sebacinales. The plectenchyma of squarrosely branched hyphae in the outer layers of *S. tomentosa* and Sebacinales specimen 6MM2 ECM is a general feature of Sebacinales ECM [50]. The transition between a dense plectenchyma of thick-walled ramified hyphae and a pseudoparenchyma in Sebacinoid ECM M2 [56] and Sebacinoid ECM Y62M1 [8], [70] was also observed in the outer mantle of *S. guayanensis*. A superficial hyphal net with variably shaped hyphae as in *S. tomentosa* and in *S. guayanensis* has also been reported from *Sebacina incrustans* + *Picea abies* and Sebacinoid ECM Y62M1 [70], *Quercirhiza dendrohyphidiomorpha* [71] and *Pinirhiza multifurcata* [72]. Thick-walled hyphae in the outer mantle layers and/or emanating elements, smooth cell walls and hyphal inflations as observed in the three species associated with *P. dipterocarpacea* are a general feature of Sebacinales ECM (Fig. 9; ref. [50]), as well as the matrix observed in *S. guayanensis* and *S. tomentosa* (Fig. 9). Polytomies observed either in the superficial hyphal net (*S. guayanensis* and *S. tomentosa*) or in the emanating hyphae (specimen 6MM2) were reported in several Sebacinales anatomotypes including Sebacinoid ECM M2 [56], Sebacinoid ECM Y62M1 and *Sebacina incrustans* + *Picea abies* [70], and *Pinirhiza multifurcata* + *Pinus tabulaeformis* [72]. The hydrophilic character of both *S. guayanensis* and *S. tomentosa*, and the dextrinoid reaction recorded in *S. guayanensis* is a common feature of Sebacinales ECM [50]. The presence of awl-shaped cystidia in specimens belonging to the *S. guayanensis* ECM anatomotype was confirmed in the present study.

The three anatomotypes discussed here belong to obviously different phylogenetic species; they belong to three different clades, and specimens belonging to the same anatomotype clustered together with a strong bootstrap support. Specimens belonging to the *S. guayanensis* ECM anatomotype were woolly, whereas they were cottony in *S. tomentosa*. *Sebacina guayanensis* was the only ECM anatomotype with yellowish cell walls. Rhizomorphs were present only in *S. guayanensis* specimens and in Sebacinales specimen 6MM2. The transition between a plectenchyma and pseudoparenchyma in *S. guayanensis* was different from the plectenchyma observed in *S. tomentosa* and Sebacinales specimen 6MM2. Hyphal walls were either thin or thicker in the outer mantle layer of *S. tomentosa*, whereas they were thick in the outer layers of the remaining two species. A matrix was present only in the outer mantle layer of *S. guayanensis* and in deeper mantle layers of *S. tomentosa*. Staghorn-like hyphae were observed only in the *S. tomentosa* ECM anatomotype. Polytomies with three to many branches were only observed in *S. tomentosa*. Clusters of emanating hyphae and frequent curved septa were only observed in specimen Sebacinales 6MM2. Awl-shaped emanating hyphae or cystidia were only present in *S. guayanensis* and Sebacinales specimen 6MM2. Specimens PD51 and 5MM2 clustered together in the *S. guayanensis* clade with a significant bootstrap support (Fig. 7). Comparisons between these specimens and the remaining *S. guayanensis* specimens suggested that fine anatomical features such as hyphal diameter and cell wall thickness might even reveal genetic differences amongst populations within the same species.

This study suggests that *S. guayanensis*, *S. tomentosa* and Sebacinales specimen 6MM2 are genetically different from strains belonging to Sebacinales species identified from fruitbodies. We cannot reject the hypothesis that the three species associated with *P. dipterocarpacea* are conspecific with Sebacinales fruitbodies that have been collected before, particularly in the neotropics, but that currently do not have a DNA record. A *Sebacina* fruitbody consistent with *S. incrustans* fruit body morphology was collected in a *Dicymbe* forest in Guyana [25], and two *Tremellodendron* species were reported from the Dominican Republic (*T. simplex*, ref. [73]) and Jamaica (*T. tenue*, ref. [74]). Taking into account the diversity of ECM Sebacinales in the Guayana region, the hypothesis that *S. guayanensis*, *S. tomentosa* and Sebacinales specimen 6MM2 are phylogenetic species that have not been described before is likely. Our analyses also confirmed that these species associated with *P. dipterocarpacea* are both genetically and anatomically different from previously described Sebacinales ECM anatomotypes. Despite similarities between *S. guayanensis* and Sebacinoid ECM Y62M1 [8], [70] regarding the mantle organization [5], *S. guayanensis* is the first reported Sebacinales ECM worldwide with yellowish cell walls. An additional important difference between the two anatomotypes is the presence of rhizomorphs only observed in *S. guayanensis*. As discussed in ref. [5], finer differences between *S. guayanensis* and Y62M1 include the greater diameter (3.5–7 versus 2.2–3.5 µm) and the different shape of hyphae in the superficial net of Y62M1, the location of polytomies, either in the net (*S. guayanensis*) or the emanating hyphae (Y62M1), and the variable diameter of emanating hyphae only in Y62M1 (1–4 versus 2.8–3.2 µm). Thick-walled, straight or wavy emanating hyphae with polytomies in Y62M1 [70] have been classified as awl-shaped (type A) or ramified (type E) cystidia [8]. Shorter emanating hyphae in *S. guayanensis* were similar to bent awl-shaped cystidia; the frequent septa and the lack of anatomical differences with longer emanating hyphae suggested they represent a transition between emanating hyphae and cystidia. Examination of specimen 5MM2 demonstrated that characteristic awl-shaped cystidia are present in ECM specimens genetically close to type specimen BM03M3. Based on an arbitrary 97% ITS similarity threshold value [10], *S. guayanensis* and undescribed Sebacinales ECM17 (JN168754) may be considered as conspecific. The phylogenetic tree (Fig. 7) indicates that specimen ECM17 is sister to the clade including *S. guayanensis* specimens. A combined anatomical and molecular phylogenetic study of specimens similar to ECM17 strain would be useful to test whether *S. guayanensis* and ECM17 are distinct, closely related species.

The present study confirmed the importance of the thick-walled, often swollen hyphae with numerous finger-like outgrowths and ramifications including polytomies with numerous branches (so-called staghorn-like hyphae) as a distinguishing taxonomic feature of *S. tomentosa*; all Sebacinales specimens with this feature belonged to a strongly supported clade. As discussed in ref. [5], cystidial types (E and I) with finger-like outgrowths have already been reported in ref. [39]. We prefer the term ‘hyphae’ for the outermost cells in the *S. tomentosa* ECM mantle because these structures were bearing a variable number of outgrowths of variable length and sometimes with septa. We designate variably shaped hyphae on the *S. tomentosa* ECM as staghorn-like hyphae for the similarity with staghorn hyphae on the *Pseudotsuga menziesii* + *Byssoporia* (*Poria*) *terrestris* var. *lilacinorosea* ECM anatomotype [8], [75]. Differences between *B. terrestris* and *S. tomentosa* staghorn-like hyphae include the thin walls as well as the lack of inflations and conspicuous polytomies in the first anatomotype.

Specimen 6MM2 belonged to the same clade as the undescribed Sebacinales strain ECM84 (JN168756). Examination of further specimens and descriptions of Sebacinales ECM84 are necessary to test whether these Sebacinales specimens belong to the same species. As mentioned in ref. [5], specimen 6MM2 shared several features with the identified ECM anatomotype *Sebacina incrustans* + *Picea abies* (sample Y129M5; refs. [8], [70]) and Sebacinaceae sp. + *Betula papyrifera* [76]. In these three anatomotypes, a matrix is lacking and the thick-walled plectenchymatous outer mantle layer is covered by numerous thick-walled emanating elements. Amongst these three anatomotypes, specimen 6MM2 is the only one with rhizomorphs. Emanating elements in *Sebacina incrustans* + *Picea abies* [8], [70] and Sebacinaceae sp. + *Betula papyrifera* [76] were interpreted as awl-shaped cystidia and emanating hyphae. The shorter emanating hyphae of specimen 6MM2 were similar to awl-shaped cystidia [50]. The thinner wall at the distal ends and the frequent occurrence of septa suggest they are a transitional growing stage of emanating hyphae.

This study confirmed that the presence of rhizomorphs is a consistent taxonomic feature of *S. guayanensis* [5] in the studied *P. dipterocarpacea* forest. An occasional presence of up to five emanating hyphae glued together for short distances of about 100 µm has been reported for *Sebacina incrustans* + *Picea abies* [70]. Infrequent type A/B rhizomorphs were recorded for *Pinirhiza nondextrinoidea* [72]. Type B rhizomorphs in *S. guayanensis* and Sebacinales specimen 6MM2 were more differentiated than those in previously described Sebacinales anatomotypes. Hyphae were usually glued together, the rhizomorphs were often thick, and they reached at least a length of 2 mm. *Sebacina guayanensis* and Sebacinales specimen 6MM2 belong to two well separated clades. The presence of rhizomorphs in phylogenetically diverse Sebacinales indicates that the capacity to form rhizomorphs might be more widespread in this fungal group than hitherto assumed [24], [50] based on descriptions of temperate specimens. Two out of three Sebacinales anatomotypes in the studied *P. dipterocarpacea* forest clearly belong to the medium distance exploration types [5].

Sebacinales illustrate well the difficulty to assign new species to a particular morphologically defined genus in a diverse fungal group documented mostly from unnamed environmental sequences [77]. None of the Sebacinales species associated with *P. dipterocarpacea* nested within well-supported clades including named Sebacinales species. The phylogenetic position of Sebacinales specimen 6MM2 suggests it may be related with *Tremelloscypha gelatinosa* from a tropical deciduous *Gymnopodium floribundum* forest in Mexico [78], but the node was not statistically significant. It is well established that strains belonging to *Sebacina* and *Tremellodendron* morphospecies are involved in ECM associations [6]. *Tremelloscypha* was considered as ECM by Tedersoo et al. [79] based on molecular phylogenetic studies [3], [51]. Bandala et al. [78] suggested that *G. floribundum* is a putative host of *T. gelatinosa* in Mexico, but the ECM status of this Sebacinales genus has not been confirmed yet. To date, a species name has been assigned to only one described Sebacinales ECM anatomotype worldwide [70], but this anatomotype belonged to the polyphyletic morphospecies complex *Sebacina incrustans* [82]. Amongst the three Sebacinales species associated with *P. dipterocarpacea*, specimen 6MM2 is closest to a *Sebacina incrustans* ECM; the presence of type B rhizomorphs in specimen 6MM2 and the tree topology demonstrate that these two anatomotypes are two different species. We propose a formal name for specimens belonging to *S. guayanensis* and *S. tomentosa*, respectively, because these taxa are monophyletic and they are characterized both genetically and anatomically [80], [81]. ECM structures are vegetative morphs during the ECM fungal life history that can be species specific. Art 8.2 of the International Code of Nomenclature for algae, fungi, and plants (ICN; ref. [83]) states that a type specimen should be a physical specimen gathered at one time, which needs not be complete. There is no restriction on the forms of type specimens (Art 8.4 in ref. [83]). Characters of vegetative cultures of fungi including a species involved in the ECM morphotype *Piceirhiza bicolorata* (see [8]) have already been demonstrated to be helpful to delineate species [84], and the genus *Meliniomyces* was typified on basis of both vegetative features of mycelium cultures and ITS data. ITS/LSU data are widely recognized as suitable barcode regions to discriminate fungal species and to infer phylogenetic relationships between taxa [85]. Using ECM samples as type specimens within Sebacinales seems appropriate for the following reasons: (i) Taxonomically informative characters can be found in ECM anatomotypes ([5], [72], this study), whereas fruiting body morphology and anatomy seem to be insufficient characteristics to separate species, at least in *Sebacina* [3], [82]; and (ii) the diversity of Group A Sebacinales ECM has predominatly been assessed based on belowground surveys. There are no clear rules to designate a taxonomic category for environmental samples [77].

A widely used ECM classification system [8] depends both on the host tree and features of the ECM. Here we generalize this system as a useful approach to describe fungal ECM species. Shared anatomical features have been found amongst Sebacinales ECM specimens belonging to the same fungal species associated with different tree hosts ([56], this study). To include both phenotypic and genetic characters, we provide both data on ECM morphology and anatomy and ITS consensus sequences in the descriptions of the two tropical Sebacinales species in the present study. *Sebacina* was selected as a generic name for species *guayanensis* and *tomentosa* because it is the type genus of the Sebacinaceae [87] and sequences of identified *Sebacina* specimens appear distributed across our phylogenetic tree (data not shown). We preferred not to assign a formal name for specimen 6MM2, before more specimens of this third anatomotype have been studied.

It is widely recognized that species should be delineated by a combination of data [81]. The need to name taxa only known from environmental sequences is an important challenge for a contemporary taxonomy [77]. This study shows that both using ECM anatomy and molecular phylogenetics may be useful taxonomic tools in Sebacinales Group A. ECM anatomy is still understudied in the highly diverse fungal group of the Sebacinales, and generalizing results from temperate areas can obviously be misleading. Anatomical features new for Sebacinales ECM such as frequent type B rhizomorphs, yellowish cell walls and staghorn-like hyphae were discovered in the Sebacinales ECM associated with *P. dipterocarpacea*. Sebacinales ECM in two well-separated, possibly only tropical clades belonged to the medium-distance exploration type. This study suggests that at least two Group A Sebacinales clades evolved separately in the tropics; distinguishing ECM anatomical features can be expected in Sebacinales species belonging to these clades. Our study highlights the importance of studying and describing tropical Sebacinales ECM to get a more complete view of Sebacinales ECM anatomotypes diversity within this broadly distributed fungal group.
