# Correction: New Neotropical Sebacinales Species from a *Pakaraimaea dipterocarpacea* Forest in the Guayana Region, Southern Venezuela: Structural Diversity and Phylogeography

**DOI:** 10.1371/journal.pone.0107078

**Published:** 2014-08-26

**Authors:** The PLOS ONE Staff

## Notice of Republication

This article was republished on August 11, 2014, to replace incorrectly changed characters in the byline, copyright statement, references, and body text in the online version only. The publisher apologizes for the errors. Please view this article again to see the correct version.
